# Night and shift work and incidence of cerebrovascular disease – a prospective cohort study of healthcare employees in Stockholm

**DOI:** 10.5271/sjweh.3986

**Published:** 2021-12-30

**Authors:** Carolina Bigert, Manzur Kader, Tomas Andersson, Jenny Selander, Theo Bodin, Per Gustavsson, Mikko Härmä, Petter Ljungman, Maria Albin

**Affiliations:** 1 Institute of Environmental Medicine, Karolinska Institutet, Stockholm, Sweden; 2 Centre for Occupational and Environmental Medicine, Region Stockholm, Stockholm, Sweden; 3 Finnish Institute of Occupational Health, Helsinki, Finland; 4 Department of Cardiology, Danderyd University Hospital, Sweden

**Keywords:** night shift, shift worker, stroke, occupational exposure, occupational health

## Abstract

**Objective:**

This study aimed to investigate the effects of various aspects of night and shift work regarding incident cerebrovascular disease (CeVD).

**Methods:**

The cohort included 26 667 women and 3793 men (nurses and nursing assistants) who were employed for at least one year 2008–2016 in Region Stockholm, Sweden. Information about the cohort and working hours were obtained from a computerized employee-register and diagnoses were retrieved from national and regional registers. We used discrete time proportional hazard models to assess the risk of CeVD (2009–2017), in relation to work hour characteristics, adjusting for sex, age, country of birth, education and profession.

**Results:**

We observed an excess risk of CeVD (N=223) among employees who, during the preceding year, worked night shifts >30 times [hazard ratio (HR) 1.44, 95% confidence interval (CI) 1.04–1.99] or ≥3 consecutive night shifts >15 times (HR 1.69, 95% CI 1.18–2.42) or with >30 quick returns (<28 hours) from night shifts (HR 1.52, 95% CI 1.10–2.10) compared to those who did not work nights. We also observed an excess risk among employees with a long duration (>5 years) of exposure to night shift work (HR 1.87, 95% CI 1.27–2.77), all supported by a dose–response pattern.

**Conclusions:**

Our results show that the risk of CeVD among nurses and nursing assistants is associated with night shift work. The number of years with night shift work, the frequency of night shifts per year, the frequency of consecutive night shifts, and short recovery after night shifts influenced the risk. Work schedules aiming at minimizing these aspects of night shift work may reduce the risk.

Cerebrovascular disease (CeVD) includes diseases that affect the cerebral blood vessels and blood supply and are mainly related to occlusion or vascular bleeding. The most common CeVD is ischemic stroke (1). Stroke is one of the leading causes of death and disability worldwide. Although the age-standardized stroke mortality rates have decreased globally over time, the absolute number of stroke occurrences and deaths have increased (2, 3).

The underlying mechanisms for CeVD may vary depending on the disease and involve an interaction of vascular risk factors, environmental and genetic factors. Many of the risk factors associated with lifestyle and environment are modifiable, such as hypertension, tobacco use, hyperlipidemia, diabetes, unhealthy dietary patterns, obesity and physical inactivity (4).

There is accumulating evidence that working time patterns like shift work, night work and long working hours may increase the risk of CeVD (5–10). Therefore, efforts to develop and modify existing work schedules for those who need to work non-standard working hours are important parts for risk management. Potential mechanisms linking night and shift work or long working hours to CeVD include disruption of the circadian rhythm, sleep deprivation, insufficient recovery, hormonal effects, stress mechanisms, inflammation, vascular effects, and metabolic disturbances (7, 9, 11–16). Several different aspects of night and shift work may possibly be important components influencing the risk of CeVD, such as the frequency of night shifts or the frequency of consecutive night shifts, related to circadian disruption, insufficient recovery and accumulated sleep debt (15). Another aspect is quick returns from work shifts, leading to sleep deprivation and an increased level of perceived stress (13). Night and shift work have also been found to be associated with poorer lifestyle behaviors and with risk factors of CeVD, such as hypertension and diabetes (17, 18), which may influence the risk. In a recent study of Swedish twins, a higher prevalence of overweight and smoking was observed among individuals who had been exposed to night shift work compared to those with no history of night work, but with no obvious difference in the prevalence of alcohol consumption or leisure-time activity (10).

Shift work is common, especially in the healthcare sector, where the proportion of shift workers (workers with daily split shifts, permanent shifts of mornings, afternoons or nights, alternating or rotating shifts, or other types of shift work) is about 40% (19). In comparison, in the European Working Conditions Survey of 2015, for all sectors combined, 21% reported shift work, 19% reported night work, 16% reported long working hours of ≥48 hours per week, and 23% reported not having time to recover (<11 hours between working days) (19). Similarly in Sweden, figures for the proportion of shift and night workers for all sectors combined were 21% and 14%, respectively (20).

In a meta-analysis of five cohort studies (21–25) a small increase in stroke mortality was reported among shift workers (7). An increased mortality in cardiovascular disease was observed among Danish nurses, who worked night shifts (8), as well as among night shift workers in a cohort of Swedish twins (10). A systematic review and meta-analysis by Torquati et al (2018) demonstrated a higher risk of cardiovascular disease events among shift workers than day workers, supported by a dose–response relationship (26).

A limitation in several of the previous studies is imprecise exposure data, with self-reported information on working hours and schedules and lack of longitudinal exposure information. This increases the risk of error in classification of exposure and prohibits the possibility to give detailed recommendations to improve the used shift patterns (6, 27, 28). Furthermore, we are not aware of any earlier studies on the association of specific shift work patterns with incident CeVD. An increased understanding of how various aspects of night and shift work (such as frequency of night shifts, consecutive night shifts and quick returns) influence the risk could contribute to knowledge on how work schedules might be optimized to reduce the risks associated with shift work.

The aim of this study was to evaluate the effects of various aspects of night and shift work, regarding incident stroke and other CeVD, by using detailed and registry-based exposure data.

## Methods

### Study population

The cohort was identified from a computerized administrative employee register (HEROMA) in Region Stockholm (formerly Stockholm County Council), Sweden. We identified healthcare employees who were employed for at least one year anytime between 1 January 2008 and 31 December 2016. The healthcare provided under the Region Stockholm’s management includes both in- and outpatient care services, such as emergency hospitals, local medical centers, family doctors and maternity clinics. We restricted the study to occupational groups with an expected high proportion of night and shift workers, mainly nurses (N=17 238) including midwives, and nursing assistants (N=13 222) including caregivers, accommodation assistants and personal assistants. Unfortunately, it was not possible to obtain precise information on working hours for physicians and therefore they were excluded in this study.

**Figure 1:**
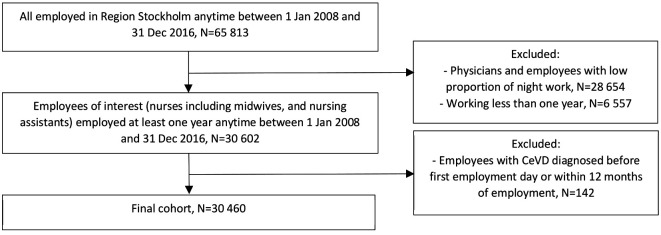
Flowchart for the inclusion and exclusion procedure, cohort for cerebrovascular disease (CeVD).

The final CeVD cohort of nurses and nursing assistants consisted of 30 460 employees, 26 667 women and 3793 men, after excluding physicians and employees in occupational groups with an expected low proportion of night work (eg, administrators, cleaners, kitchen assistants, psychologists and physiotherapists) and employees with any CeVD before first employment day (based on data back to 1998) or within the first year of employment. A flowchart for the inclusion and exclusion procedure is presented in figure 1.

### Data sources and assessment of the outcome

The information on working hours was obtained from the same computerized administrative employee register (HEROMA) as for the identification of the cohort. From the HEROMA system we collected detailed individual information on working hours day-by-day for all employed from 1 January 2008 to 31 December 2016, including information on occupation and workplace for each working period. For each work shift, there was information on the exact start and end time. Information about education and country of birth was collected from the register of the total population at Statistics Sweden.

Information on the outcome CeVD (I60-69 in ICD-10) was retrieved from the national patient register at The National Board of Health and Welfare (inpatient care) for the period 2009–2016 and from the regional database (VAL database) for the period 2009–2017. The VAL database includes diagnoses from both inpatient care and outpatient contacts (health centers, emergency services and hospital visits where the patients have not been hospitalized) within the Region Stockholm. We included the CeVD diagnosis that appeared first in either of the two registers, during the follow-up period 1 January 2009 to 31 December 2016 (N=201), and for the period 1 January 2017 to 31 December 2017 information was available only from the VAL database (N=22). We used incident stroke (I61, I63 and I64 in ICD-10) as a separate outcome. The employees were at risk from one year after the start of employment until CeVD diagnosis, death, or end-of-follow-up, whichever came first.

### Classification of exposure

We aggregated the information on working hours from HEROMA to classify persons with respect to different types of shift work and working hours. Shifts <4 hours were not included. The method for classifying working time patterns based on payroll-based daily objective working hours was first evaluated in the Finnish healthcare system (27), and the method for aggregation on working hours from HEROMA was recently described in a study based on the same cohort of healthcare employees as in the present study, but with a focus on shift and night work during pregnancy and risk of preterm birth (29).

All shifts were classified as follows: (i) Daytime work only (starts after 06:00 and ends no later than 18:00 hours), (ii) afternoon shift (starts after 12:00 and ends later than 18:00 hours but not a night shift), (iii) night shift (≥3 hours between 22:00–06:00 hours, based on Swedish Working Hours Act). Based on this classification of shifts, the cohort subjects were, for each year, classified as (i) daytime workers, (ii) persons also working afternoon shifts but not night shifts, (iii) persons working various shift types including night shifts, or (iv) persons working night shifts only. The exposure classification was assessed annually to take into account variations over time.

Among all employees, for each year, we defined the frequency of night shifts per year (0, 1–30, >30 times), the frequency of ≥3 consecutive night shifts per year (0, 1–15, >15 times) quick returns from night shifts (<28 hours between the end of the night shift and the beginning of the following shift) or from other shifts (<11 hours) per year (0, 1–30, >30 times), and the frequency of long (>45 hours) working weeks per year (0, 1–10, >10 times). We also classified the cumulative number of years with night shifts since 2008 (0, 1–5, >5 years). The cut off points for the aspects of night and shift work and long working weeks were chosen on the basis of a compromise between obtaining a sufficiently large contrast between the exposure categories while not losing power. However, we also conducted analyses based on continuous measures (test for trend).

### Data analysis

To assess the effect of different types of shift work and working hours on the risk of CeVD, we used discrete time proportional hazard models, stratifying the person-time experience of the cohort by follow-up year, starting the assessment of risk one year after first employment day (30, 31). In the estimations of hazard ratios (HR), we adjusted for sex, age (continuous), country of birth (Sweden; Nordic countries except Sweden; Europe except Nordic countries; other countries), education (higher education (university ≥3 years); upper secondary or elementary school or less), and profession (nurses including midwives; nursing assistants including caregivers, accommodation assistants and personal assistants). We adjusted for country of birth since there are differences in CeVD risk with ethnicity, and adjustments for educational level and profession were used as a proxy for socioeconomic status that also affects the risk. Age and exposure variables were treated as time-dependent variables updated for each year of follow-up, whereas sex, country of birth, education and profession were fixed. Each of the models per disease was estimated separately. In additional analyses, we also adjusted for the total number of night shifts per year among employees who worked at least one night shift during the year in order to differentiate between the effect of the intensity of night work in general and the effect of different aspects of night shift work such as frequently working consecutive night shifts or having quick returns from night shifts.

We estimated HR with 95% confidence intervals (CI) for CeVD in relation to different aspects of night and shift work (eg, type and frequency of shift work, consecutive shifts, and quick returns) and frequency of long working weeks. For type of shift work, the comparisons were performed with those who always worked day shifts as the reference group. For the other exposure variables, the comparisons were performed with those who worked day and/or afternoon shifts but no night shifts (’never night shifts’) or with the lowest exposure category as the reference group. The estimated HR were based on the exposure during the year preceding the outcome, except for analyses of cumulative years of night work.

The test for trend with number of times per year of night shifts and the number of times per year of the different aspects of night and shift work and long working weeks used the arithmetic average of number of times in each exposure category as a continuous variable in the regression model. The test for trend with cumulative number of years with night shifts used the arithmetic average of number of night-years in each exposure category as a continuous variable.

In a sensitivity analysis, we restricted the analysis to employees who ever worked ≥3 consecutive night shifts in order to explore the effect of different levels of exposure to consecutive night shift work from the effect of general night work. Comparisons were then performed with those with the lowest exposure category of consecutive night shift work as the reference group.

In additional analyses, we analyzed the risk of CeVD among women and men separately.

We used SAS software version 9.4 for Windows (SAS Institute Inc., Cary, NC, USA) for all statistical analyses.

## Results

The baseline characteristics (sex, age, education, country of birth and profession) of the study participants at the inclusion year, by work schedule, are presented in table 1. The baseline characteristics are from the inclusion year but the work schedules are based on all the years the person worked during 2008–2016. There were 6883 employees with permanent daytime work during the whole employment period, 11 192 with shift work but no night shifts, 11 250 with shift work including night shifts (but not only night shifts) and 1135 with only night shifts during the whole employment period. The proportion of younger employees (≤40 years) at the inclusion year was higher among shift workers and night workers who did not work only nights (shift workers without night shifts: 55%, shift workers with night shifts but not only nights: 65%) than among employees with permanent daytime work (25%) or permanent night work (23%) during the whole employment period. The proportion of men as well as employees with country of birth outside the Nordic countries was higher in the two shift work groups and among night workers. The proportion of employees with higher education as well as nurses was lowest (29% and 33%, respectively) among employees who only worked night shifts during the whole employment period.

**Table 1:** Baseline characteristics (sex, age, education, country of birth, profession) of the study participants (N=30 460) at the inclusion year in the cohort for all cerebrovascular diseases (ICD: I60-I69). Subdivided by work schedule based on all the years the person worked during 2008–2016.

Variables	Day work only ^a^ (N=6883)		Shift work, without night shifts ^b^ (N=11 192)		Shift work, with night shifts ^c^ (N=11 250)		Night work only ^d^ (N=1135)	
			
	N	%	N	%	N	%	N	%
Sex								
Women	6449	94	9748	87	9631	86	839	74
Men	434	6	1444	13	1619	14	296	26
Age (years)								
≤40	1725	25	6195	55	7349	65	260	23
41–50	2098	31	2645	24	2450	22	351	31
>50	3060	44	2352	21	1451	13	524	46
Education								
Higher education (university ≥3 years)	3810	55	5647	50	7424	66	335	29
Upper secondary, elementary or less	2933	43	5343	48	3629	32	747	66
Missing	140	2	202	2	197	2	53	5
Country of birth								
Sweden	5715	83	8599	77	8685	77	811	71
Nordic countries (except Sweden)	529	8	503	4	547	5	113	10
Europe (except Nordic countries)	129	2	344	3	402	4	47	4
Other countries	510	7	1746	16	1616	14	164	15
Profession								
, including midwives	4760	69	5135	46	6969	62	374	33
Nursing assistants ^e^	2123	31	6057	54	4281	38	761	67

a Day work: starts after 06:00 and ends no later than 18:00 hours.

b At least one afternoon shift (starts after 12:00 and ends later than 18:00 hours, but not a night shift).

c At least one night shift (≥3 hours between 22:00–06:00 hours), but not only night work.

d Only night work (no day work or afternoon shifts).

e Assistant nurses, caregivers, accommodation assistants and personal assistants.

The 30 460 employees contributed a total of 240 469 person-years. During the follow-up (up to 9 years, mean 4.7 years), there were 223 incident cases of CeVD in the cohort. Of these, 162 cases were first identified from the national patient register and 61 cases from the regional VAL database. The 223 incident CeVD cases included in total 132 cases of stroke of which 115 were ischemic stroke, 16 were hemorrhagic stroke and one was an unspecified cerebral infarction.

### Type and frequency of night work

We found that healthcare employees with shift work including night shifts and employees who only worked night shifts tended to have an increased risk of CeVD the following year compared to those with only day work, although not statistically significant (HR 1.46, 95% CI 0.96–2.21 and HR 1.39, 95% CI 0.88–2.21, respectively) (table 2). The risk was more pronounced among employees who frequently worked night shifts (>30 times per year: HR 1.44, 95% CI 1.04–1.99), and who frequently (>15 times per year) worked ≥3 consecutive night shifts (HR 1.69, 95% CI 1.18–2.42) compared to those who never worked night shifts (table 2). There was a trend of increasing risk estimates with increasing number of times of night shifts (HR for beta 1.003, 95% CI 1.000-1.006, ie, the risk increased by 0.3% for each additional night shift), and for increasing number of times of ≥3 consecutive night shifts (HR for beta 1.018, 95% CI 1.008–1.031, ie, the risk increased by 1.8% for each additional spell of ≥3 consecutive night shifts) (table 2). The association remained for ≥3 consecutive night shifts after additional adjustment for the total number of night shifts, with higher risk estimates and a higher risk increase per time but with wider CI (table 3). In a sensitivity analysis restricting the analyses to employees who ever worked ≥3 consecutive night shifts, the risk was 1.44 (95% CI 0.86–2.43) for employees who frequently (>15 times per year) worked ≥3 consecutive night shifts, compared to those with a frequency of 1–15 times of ≥3 consecutive night shifts per year (data not shown).

**Table 2:** Discrete-time proportional adjusted ^a^ hazard ratios (HR) with 95% confidence intervals (CI) for first diagnosed cerebrovascular disease (ICD- 10: I60-I69, N=223) and stroke (ICD-10: I61+I63+I64, N=132), among healthcare employees (N=30 460) during follow-up 2009–2017, contributing to a total of 240 469 and 241 197 person-years (PY), respectively. Each of the models per disease was estimated separately.

Exposure ^b^	Cerebrovascular disease, all				Stroke			
	
	PY	No of cases	Cases per 10 000 PY	HR (95% CI)	PY	No of cases	Cases per 10 000 PY	HR (95% CI)
Type of shift work								
Always day shifts	80 662	83	10.3	Ref.	81 001	50	6.2	Ref.
Day and/or afternoon shifts (no nights)	93 771	77	8.2	1.21 (0.86–1.69)	94 040	45	4.8	1.15 (0.75–1.77)
Day and/or afternoon shifts, and nights	48 129	34	7.1	1.46 (0.96–2.21)	48 191	20	4.2	1.37 (0.79–2.36)
Night shifts only	17 888	29	16.2	1.39 (0.88–2.21)	17 946	17	9.5	1.23 (0.68–2.25)
Frequency of night shifts								
Never night shifts ^c^	174 458	160	9.2	Ref.	175 066	95	5.4	Ref.
1–30 (mean 12.6) times	22 983	10	4.4	1.03 (0.54–1.97)	23 000	4	1.7	0.61 (0.22–1.68)
>30 (mean 104.0) times	43 028	53	12.3	1.44 (1.04–1.99)	43 131	33	7.7	1.35 (0.89–2.05)
Trend test (risk increase per time)				1.003 (1.000–1.006)				1.003 (0.999–1.007)
Frequency of ≥3 consecutive nights								
Never night shifts ^c^	178 543	160	9.0	Ref.	179 156	95	5.3	Ref.
1–15 (mean 5.3) times	31 964	18	5.6	1.20 (0.74–1.94)	32 017	10	3.1	1.05 (0.54–2.03)
>15 (mean 27.8) times	24 783	38	15.3	1.69 (1.18–2.42)	24 836	24	9.7	1.60 (1.01–2.54)
Trend test (risk increase per time)				1.018 (1.008–1.031)				1.017 (1.008–1.034)
Quick returns (<28 h) from nights								
Never night shifts ^c^	175 262	160	9.1	Ref.	175 871	95	5.4	Ref.
1–30 (mean 8.3) times	24 675	16	6.5	0.83 (0.42–1.64)	24 693	7	2.8	0.65 (0.24–1.79)
>30 (mean 64.2) times	39 894	47	11.8	1.52 (1.10–2.10)	39 995	30	7.5	1.42 (0.93–2.15)
Trend test (risk increase per time)				1.006 (1.001–1.011)				1.006 (0.999–1.012)
Quick returns (<11 h) from other shifts ^d^								
Never quick returns from other shifts	91 707	99	10.8	Ref.	92 113	61	6.6	Ref.
1–30 (mean 13.9) times	33 390	25	7.5	1.13 (0.78–1.63)	33 471	12	3.6	1.03 (0.63–1.69)
>30 (mean 55.2) times	50 165	36	7.2	0.85 (0.59–1.23)	50 287	22	4.4	0.85 (0.53–1.37)
Trend test (risk increase per time)				0.996 (0.989–1.002)				0.995 (0.987–1.004)
Cumulative number of years with nights								
Never	153 921	143	9.3	Ref	154 446	88	5.7	Ref
1–5 (mean 2.6) years	62 809	42	6.7	1.17 (0.82–1.68)	62 939	19	3.0	0.84 (0.50–1.41)
>5 (mean 7.7) years	23 739	38	16.0	1.87 (1.27–2.77)	23 812	25	10.5	2.04 (1.26–3.28)
Trend test (risk increase per year)				1.083 (1.031–1.139)				1.088 (1.021–1.159)
Frequency of long (>45 h) working weeks								
Never (only working weeks of ≤40 h)	93 429	90	9.6	Ref.	93 846	50	5.3	Ref.
1–10 (mean 4.0) times	96 860	64	6.6	1.23 (0.89–1.69)	97 061	37	3.8	1.38 (0.91–2.08)
>10 (mean 14.6) times	30 133	25	8.3	1.06 (0.66–1.68)	30 195	13	4.3	0.99 (0.52–1.87)
Trend test (risk increase per time)				1.005 (0.975–1.036)				1.002 (0.963–1.043)

a Adjusted for sex, age (continuous), country of birth (Sweden; Nordic countries except Sweden; Europe except Nordic countries; other countries), education (higher education; upper secondary, elementary school or less) and profession (Nurses including midwives; Nursing assistants). Information on education was missing for 1.9% of the participants (no imputation was used to represent missing values).

b Based on the exposure during the year preceding the outcome, except for analyses of cumulative exposure.

c Those who worked day and/or afternoon shifts but no night shifts.

d Analyses were based on those who never worked night.

For the more specific outcome incident stroke, we found similar trends and a statistically significant association among employees who frequently (>15 times per year) worked ≥3 consecutive night shifts (table 2). In the sensitivity analysis restricted to employees who ever worked ≥3 consecutive night shifts, the risk of stroke was 1.66 (95% CI 0.81–3.37) for employees who frequently (>15 times per year) worked ≥3 consecutive night shifts, compared to those with a frequency of 1–15 times of ≥3 consecutive night shifts per year (data not shown).

### Quick returns

We observed that employees who often (>30 times per year) had quick returns (<28 hours) from night shifts had an increased risk of CeVD (HR 1.52, 95% CI 1.10–2.10) the following year compared to those who never worked night shifts. There was a trend of increasing risk estimates with increasing number of times of quick returns from night shifts (HR for beta 1.006, 95% CI 1.001–1.011, ie, the risk increased by 0.6% for each additional quick return) (table 2). The association remained for quick returns from night shifts after additional adjustment for the total number of night shifts, with higher risk estimates and a higher risk increase per time but with wider CI (table 3). Quick returns (<11 hours) from other shifts did not increase the CeVD risk (table 2). Associations with incident stroke demonstrated similar trends for quick returns from night shifts although not statistically significant (table 2).

**Table 3:** Discrete-time proportional adjusted ^a^ hazard ratios (HR) with 95% confidence intervals (CI) for first diagnosed cerebrovascular disease (ICD-10: I60-I69, N=223) and stroke (ICD-10: I61+I63+I64, N=132), among healthcare employees (N=30 460) during follow-up 2009-2017, contributing to a total of 240 469 and 241 197 personyears (PY), respectively. Additionally adjusted for the total number of night shifts per year.

Exposure ^b^	Cerebrovascular disease, all	Stroke
	
	HR (95% CI)	HR (95% CI)
Frequency of ≥3 consecutive nights		
Never nights ^c^	Ref.	Ref.
1–15 (mean 5.3) times	1.92 (1.04–3.54)	1.61 (0.69–3.76)
>15 (mean 27.8) times	5.54 (1.83–16.76)	4.40 (1.00–19.32)
Trend test (risk increase per time)	1.053 (1.013–1.095)	1.048 (0.996–1.102)
Quick returns (<28 h) from nights		
Never nights ^c^	Ref.	Ref.
1–30 (mean 8.3) times	0.94 (0.47–1.87)	0.72 (0.26–2.02)
>30 (mean 64.2) times	3.09 (1.26–7.56)	2.64 (0.80–8.72)
Trend test (risk increase per time)	1.015 (1.002–1.029)	1.013 (0.995–1.032)

a Adjusted for sex, age (continuous), country of birth (Sweden; Nordic countries except Sweden; Europe except Nordic countries; other countries), education (higher education; upper secondary, elementary school or less), profession (Nurses including midwives; Nursing assistants) and the total number of night shifts per year. Information on education was missing for 1.9% of the participants (no imputation was used to represent missing values).

b Based on the exposure during the year preceding the outcome.

c Those who worked day and/or afternoon shifts but no night shifts.

### Cumulative number of years with night shifts

For cumulative night work, the analyses were based on exposure information since 2008. We observed an excess risk of CeVD among employees who had worked night shifts for >5 years since 2008 (HR 1.87, 95% CI 1.27–2.77), and there was a trend of increasing risk estimates with increasing number of years of night shift work (HR for beta 1.083, 95% CI 1.031–1.139, ie, the risk increased by 8.3% for each additional year of night work) (table 2). The excess risk among employees with >5 years of night work was also demonstrated in separate analyses of stroke (HR 2.04, 95% CI 1.26–3.28; HR for beta 1.088, 95% CI 1.021–1.159, ie, the risk increased by 8.8% for each additional year of night work) (table 2).

### Frequency of long working weeks

We did not observe an increased risk of CeVD or stroke among employees who often (>10 times per year) had long working weeks of >45 hours, compared to those with working weeks of ≤40 hours, and there was no trend of increasing risk estimates with increasing number of times of long working weeks (table 2). The statistical power was too low to study the risk of CeVD in association with the frequency of longer working weeks (only 4 cases among employees who often (>10 times per year) had long working weeks of >50 hours).

### Sex-related risks

Stratified analyses of the CeVD risk among women (N=26 667) and men (N=3793) separately did not add much information since there were relatively few men in the cohort, generating few cases (36 CeVD cases among men, 187 CeVD cases among women). The results for women separately were thus very similar to those for the total cohort, although with a tendency of slightly higher risks for women than men, and for men the analyses resulted in low numbers and low statistical power (supplementary material, table S1, https://www.sjweh.fi/article/3986).

## Discussion

This is a cohort study of Swedish healthcare employees where we used registry-based assessed day-by-day exposure information on working-hours to investigate the association between different types of night shift work and frequency of long working weeks and incident CeVD. We also investigated the risk of stroke. Our main findings were that employees who worked night shifts >30 times per year or who worked ≥3 consecutive night shifts >15 times per year, had an excess risk of CeVD the following year compared to those with no night work. There was also an excess CeVD risk among employees with >30 quick returns from night shifts per year and among employees with a cumulative exposure of >5 years of night work. We observed a trend of increasing risk estimates for CeVD with increasing number of times of night shifts, times of ≥3 consecutive night shifts, times of quick returns from night shifts and the number of years of night shift work. Long working weeks of >45 hours >10 times per year did not increase the risk.

Analyses specifically targeting stroke demonstrated similar risks to those for total CeVD. The risk of incident stroke was statistically significantly increased among employees who frequently (>15 times per year) worked ≥3 consecutive night shifts and among employees with >5 years of night work, supported by a dose–response pattern.

Since the exposure variables frequency of ≥3 consecutive night shifts and frequency of quick returns from night shifts are correlated to the number of night shifts worked, we performed additional analyses that also adjusted for the total number of night shifts per year. The effect from the different aspects of night shift work was stronger after this adjustment and thus the effect of working consecutive night shifts and having quick returns from night shifts seems to be at least partially separated from the effect of the intensity of night work in general.

We are not aware of any earlier studies on the association of the specific characteristics of shift work – like the intensity of night shifts or quick returns – with CeVD. However, our results are in line with previous reviews and meta-analyses reporting an increased risk of stroke among shift workers. Vyas et al (5) defined shift work as all schedules that were not regular daytime work, also including night work. In the pooled analyses they demonstrated an increased risk of ischemic stroke (based on two studies) and a non-significant increase of CeVD mortality (based on four studies) among shift workers, compared to non-shift workers. Li et al (7) included five cohort studies [of which two were also included in the study by Vyas et al (5)] in a meta-analysis and observed a slightly increased stroke mortality. In a prospective cohort study of Swedish twins, the risk of mortality due to cardiovascular disease (including stroke) was increased among night shift workers, compared to those with no night work, after adjustment for several important risk factors (such as smoking, alcohol consumption, body mass index and physical activity at leisure time) and with higher risk estimates in night workers with a work duration of >5 years (10).

Our findings indicate both subacute effects (within a year of night shift work) and long-term effects (after many years of night shift work) on CeVD risk. All the analyses, except for cumulative number of years with night work, focused on the risk associated with the exposure during the previous year. Since the exposure information was updated yearly, it is possible for participants to be in, eg, the day and/or afternoon shift group one year and the night shift group another year and vice versa. Our results are thus based on a narrow time window for the exposure (recent exposure) and do not exclude that the participants may have belonged to other exposure categories in previous years.

Based on our study, we cannot determine the underlying mechanisms of the increased risk, although a disturbed circadian rhythm and consequent effects thereof are likely to have an impact. There is previous evidence of an effect on hormones such as cortisol and melatonin from exposure to consecutive night shifts (32–34). Associations have also been observed between recent night and rotational work and the incidence of hypertension (17) (a common risk factor for both ischemic and hemorrhagic stroke), and between shift work and increased use of medications for type-2-diabetes, dyslipidemia and hypertension (18). Sleep deprivation and insufficient recovery, associated to consecutive night shifts and quick returns, could be a possible pathway from night shift work to the increased CeVD risk. Sleep duration is reduced after nights shifts and does not increase with more consecutive night shifts, leading thus to accumulated sleep debt (15). Insufficient time between the shifts (<11 hours) are associated with short sleep duration, increased sleepiness and higher level of perceived stress (13), as well as increased prevalence of shift work sleep disorder (16), although in the present study we only observed an association between CeVD risk and quick returns from night shifts but not from other shifts. Short sleep and insomnia increase the risk for coronary heart disease (35, 36) in some prospective studies, and are associated with several CeVD risk factors like obesity and diabetes (37, 38).

We also found that insufficient recovery after night shifts (<28 hours) was associated with an increased risk of CeVD. Employees, who perceived incomplete recovery from work during free weekends had an elevated risk of cardiovascular death in a 26-year follow-up (39). Disturbed sleep could even be a common pathway connecting both night and shift work, extended workhours, and work stress to adverse cardiovascular health (11).

We did not find an association between frequent (>10 times per year) long working weeks (>45 hours) and CeVD risk in this cohort, but our study does not rule out an elevation of CeVD risk of frequent very long working weeks of >50 hours. Other studies have found evidence for a relation between average long weekly working hours of ≥55 hours and incident stroke, compared to those who worked 35–40 hours per week (6, 9).

### Strengths and limitations

The major strengths of this study include the large and prospective cohort dataset and the objective exposure data from employee registers. The exposure data is very detailed with individual day-to-day information on different types of shift work and working hours. No data needed to be obtained from the employees themselves, which eliminates the risk that people will forget details about their working hours back in time. Also, the so-called “recall bias” can be avoided since the classification of exposure is not adversely affected by the outcome. Other strengths are that the cohort entirely consists of employees within the healthcare sector, where the percentage of night shift workers is high, and that the data on health outcomes are retrieved from registers of good quality. Another advantage is that we were able to collect information on cases not only from hospital care but also from outpatient visits, ie, we did not miss cases among those who have only received outpatient treatment.

A limitation of the study is that we do not have information on important individual risk factors for CeVD, such as smoking habits and other lifestyle factors, or information on work stressors, such as job strain. These could be confounders or mediators. However, the study is conducted on a cohort of nurses and nursing assistants, which is a more homogenous socioeconomic group than the population as a whole. Therefore, the potential differences in lifestyle risk factors and job strain are probably not so pronounced.

There is also a risk of underestimating the negative effects of night work by the so-called “healthy worker effect” among employees who are working night shifts. Such an effect could, for example, occur if employees with health problems already at the time of entering working life choose not to work at night or if employees are transferred from night work to day work due to a medical condition. In Sweden there is a legal requirement that employers must offer medical checks for night workers at the start of the employment and then regularly, although it is not always complied with (40). However, there may also be a selection into night shift work of employees with unfavorable risk factor profiles (41). Due to the medical checks, the “healthy worker effect” among night workers is probably stronger than the selection into night work of employees with unfavorable risk factor profiles, and therefore our findings might possibly underestimate the actual risk.

We made additional analyses for men and women separately. However, the relatively low proportion of men (12.5%) in the cohort reduced the statistical power to detect an effect among men separately. For women, the effect was similar as for the total cohort.

### Concluding remarks

In conclusion, using registry-based day-by-day exposure information in this cohort of 30 460 Swedish healthcare employees, we have shown that the risk of CeVD among nurses and nursing assistants is associated with night shift work. The number of years with night shift work, the frequency of night shifts per year, the frequency of consecutive night shifts, and time for recovery after night shifts influenced the risk. We believe that work schedules aiming at minimizing these aspects of night shift work may reduce the risk. However, it is important that advice on preventive measures includes also protection against other potential adverse health effects of night and shift work, such as eg, heart disease, cancer, adverse effects on pregnancy outcome and work-related accidents. We hope that future research in this area can contribute information as a basis for overall advice that covers other potential health effects as well.

## Supplementary material

Supplementary table
