# Supplementary material for: Night and shift work and incidence of cerebrovascular disease – a prospective cohort study of healthcare employees in Stockholm
Source: Scand J Work Environ Health. 2021 Dec 30;48(1):31–40. doi: 10.5271/sjweh.3986 (PMC8729165; doi:10.5271/sjweh.3986)
Supplement: Supplementary table [file SJWEH-48-31-S001.pdf]

Night and shift work and incidence of cerebrovascular disease – a prospective cohort study of healthcare employees in Stockholm[1]

by Carolina Bigert, MD,[2] Manzur Kader, PhD, Tomas Andersson, BSc, Jenny Selander, PhD, Theo Bodin, MD, Per Gustavsson, MD, Mikko Härmä, MD, Petter Ljungman, MD, Maria Albin, MD

1. *Supplementary table*
2. *Correspondence to: Carolina Bigert, Institute of Environmental Medicine, Karolinska Institutet, Solnavägen 4 10th floor, SE-113 65 Stockholm, Sweden. [E-mail: carolina.bigert@ki.se]*

Supplementary table 1: Discrete-time proportional adjusted[a] hazard ratios (HR) with 95% confidence intervals (CI) for first diagnosed Cerebrovascular disease (ICD-10: I60-I69; n=187 for women, n=36 for men) among female (N=26 667) and male (N=3 793) health care employees during follow-up 2009-2017, contributing to a total of 211 338 and 29 131 person-years (PY), respectively. Each of the models per disease was estimated separately.

	Cerebrovascular disease, women				Cerebrovascular disease, men			
Exposure[b]	PY	No of cases	Cases per 10 000 PY	HR (95% CI)	PY	No of cases	Cases per 10 000 PY	HR (95% CI)
Type of shift work								
Always day shifts	74 878	74	9.9	Ref.	5 784	7	12.1	Ref.
Day and/or afternoon shifts (no nights)	81 482	60	7.4	1.23 (0.85-1.78)	12 289	17	13.8	1.40 (0.56-3.50)
Day and/or afternoon shifts, and nights	41 081	32	7.8	1.62 (1.05-2.52)	7 048	4	5.7	0.89 (0.25-3.11)
Nights shifts only	13 887	21	15.1	1.43 (0.84-2.42)	4 001	8	20.0	1.45 (0.51-4.13)
Frequency of night shifts								
Never night shifts[c]	156 375	135	8.6	Ref.	18 084	24	13.3	Ref.
1-30 (mean 12.6) times	19 697	9	4.6	1.17 (0.59-2.31)	3 286	1	3.0	0.47 (0.06-3.58)
>30 (mean 104.0) times	35 266	43	12.2	1.47 (1.02-2.10)	7 761	11	14.2	1.05 (0.51-2.14)
Trend test (risk increase per time)				1.004 (1.000-1.007)				1.001 (0.994-1.007)
Frequency of ≥3 consecutive nights								
Never night shifts[c]	160 122	135	8.4	Ref.	18 421	24	13.0	Ref.
1-15 (mean 5.3) times	26 661	20	7.5	1.33 (0.80-2.22)	5 303	2	3.8	0.45 (0.10-1.92)
>15 (mean 27.8) times	19 999	27	13.5	1.66 (1.10-2.51)	4 781	8	16.7	1.38 (0.65-2.90)

Trend test (risk increase per time)				1.018 (1.004-1.033)				1.013 (0.985-1.041)
Quick returns (<28 h) from nights								
Never night shifts[c]	157 110	135	8.6	Ref.	18 152	24	13.2	Ref.
1-30 (mean 8.3) times	21 082	9	4.3	0.92 (0.45-1.90)	3 593	1	2.8	0.40 (0.05-3.04)
>30 (mean 64.2) times	32 609	43	13.2	1.55 (1.08-2.22)	7 285	11	15.1	1.10 (0.54-2.25)
Trend test (risk increase per time)				1.007 (1.001-1.013)				1.002 (0.991-1.013)
Quick returns (<11 h) from other shifts[d]								
Never quick returns from other shifts	83 459	86	10.3	Ref.	8 248	13	15.8	Ref.
1-30 (mean 13.9 times)	29 669	18	6.1	1.01 (0.59-1.72)	3 721	6	16.1	1.40 (0.52-3.79)
>30 (mean 55.2 times)	43 982	31	7.0	1.08 (0.69-1.69)	6 183	5	8.1	0.55 (0.19-1.59)
Trend test (risk increase per time)				1.001 (0.994-1.009)				0.990 (0.973-1.006)
Cumulative number of years with nights								
Never	138 047	120	8.7	Ref	15 874	23	14.5	Ref
1-5 (mean 2.6) years	53 757	35	6.5	1.38 (0.92-2.06)	9 052	7	7.7	1.26 (0.88-1.82)
>5 (mean 7.7) years	19 534	32	16.4	1.59 (1.03-2.45)	4 205	6	14.3	1.49 (1.00-2.21)
Trend test (risk increase per year)				1.092 (1.035-1.153)				1.011 (0.896-1.141)
Frequency of long (>45 h) working weeks								
Never (only working weeks of ≤40 h)	85 907	80	9.3	Ref.	7 516	10	13.3	Ref.
1-10 (mean 4.0) times	83 140	48	5.8	1.16 (0.82-1.65)	13 705	16	11.7	1.49 (0.68-3.29)
>10 (mean 14.6) times	24 804	20	8.1	1.23 (0.73-2.06)	5 329	5	9.4	0.81 (0.28-2.41)
Trend test (risk increase per time)				1.014 (0.979-1.049)				0.989 (0.927-1.055)

[a]Adjusted for age (continuous), country of birth (Sweden; Nordic countries except Sweden; Europe except Nordic countries; other countries), education (higher education; upper secondary, elementary school or less) and profession (Nurses including midwives; Nursing assistants). Information on education was missing for 1.9% of the participants (no imputation was used to represent missing values).
[b]Based on the exposure during the year preceding the outcome, except for analyses of cumulative exposure.
[c]Those who worked day and/or afternoon shifts but no night shifts.
[d]Analyses were based on those who never worked night.
